# Predicting Carotid Body Tumors’ Hardness via Multimodal Imaging: A Retrospective Cohort Study

**DOI:** 10.3390/diagnostics16121852

**Published:** 2026-06-15

**Authors:** Jiazhi Yu, Kangxi Cao, Guangnan Ao, Yunfeng Han, Tao Wang

**Affiliations:** Department of Neurosurgery, Peking University Third Hospital, Beijing 100191, China; pkuhscyujiazhi@pku.edu.cn (J.Y.); caokangxi@hsc.pku.edu.cn (K.C.); aoguangnan132@163.com (G.A.)

**Keywords:** carotid body tumors, multimodal imaging, tumor hardness, surgical complexity, imaging biomarker

## Abstract

**Background**: Carotid body tumors (CBTs) are rare neuroendocrine neoplasms whose hardness (soft vs. hard) correlates with surgical complexity and perioperative complications. This study aimed to identify predictive multimodal imaging biomarkers of CBTs’ hardness. **Methods**: This single-center retrospective cohort study included 82 patients with CBTs who underwent surgical resection. Preoperative multimodal imaging and clinical data were analyzed; tumor hardness was assessed via Masson-stained fibrous proportion. Multivariate logistic regression was performed to identify independent predictors. **Results**: The mean age of the 82 patients was 46 ± 13 years, including 37 males, with no significant intergroup differences in age or gender. Hard CBTs were associated with longer operative durations and a higher incidence of perioperative complications including pre-, intra-, and postoperative nerve and vascular injury. Multimodal imaging analysis revealed differences in signal homogeneity on T1WI and T1WI-CE sequences of MRI between soft and hard CBTs. The CBT-to-sternocleidomastoid muscle (SCM) value on T2WI (OR 0.329; 95% CI 0.151–0.591, *p* < 0.001) and the erosion of perivascular fat space (PFS) (OR 19.2; 95% CI 4.390–115.884, *p* < 0.001) were associated with the hardness of CBTs. ROC curve analysis demonstrated that an optimal cutoff value of 2.44 for the CBT/SCM ratio on T2WI predicted hard CBTs with a specificity of 100% and a sensitivity of 67.7% (PPV 100%, NPV 83.6%, AUC = 0.892). **Conclusions**: Preliminary findings suggest that CBT/SCM value on T2WI and PFS erosion are promising imaging biomarkers for predicting hardness. These parameters may facilitate preoperative risk prediction, though further prospective validation is required.

## 1. Introduction

Carotid body tumors (CBTs) are neuroendocrine paragangliomas with varying malignant potential that arise from the carotid body at the carotid artery bifurcation, with a reported incidence rate of 1–2/100,000 [1]. CBTs typically present as asymptomatic, gradually enlarging neck masses [2]. These tumors can compress and encroach upon adjacent structures, potentially leading to symptoms like hoarseness, chronic pain, dysphagia, Horner’s syndrome, transient ischemic attacks, and vertigo [3]. While surgical resection remains the standard treatment, the unique anatomical location of CBTs presents significant surgical challenges, increasing the risk of neurovascular injury and subsequent severe complications [4]. Our clinical experience, corroborated by that of other researchers, has demonstrated that the hardness of CBTs is closely associated with the surgical complexity and the incidence of perioperative complications [5,6].

Historically, preoperative evaluation of CBTs has heavily relied on morphological classifications, most notably the Shamblin system [7], to assess vascular envelopment and predict surgical morbidity. While the Shamblin classification is invaluable for estimating vascular relationships, it does not account for intrinsic tumor hardness, which independently influences surgical difficulty. Therefore, new imaging biomarkers of tumor hardness are intended to complement the Shamblin system. This combined approach may help partially overcome the limitations of purely morphological assessments, offering a more comprehensive prediction of surgical complexity. Although high-resolution MRI (hr-MRI) and contrast-enhanced sequences can successfully elucidate anatomical relationships and tumor enhancement patterns, no structured multimodal imaging criteria currently exist to preoperatively define CBT hardness. To address this clinical gap, we hypothesized that specific intrinsic and extrinsic features derived from multimodal imaging—such as internal signal intensity ratios on MRI and patterns of perivascular tissue invasion—differ significantly between soft and hard CBTs and can therefore serve as reliable preoperative predictors. Accordingly, the aim of this study is to explore the relationship between tumor hardness and multimodal imaging features, thereby identifying non-invasive biomarkers predictive of tumor hardness and surgical complexity.

## 2. Materials and Methods

### 2.1. Study Design and Participants

This single-center retrospective cohort study included patients diagnosed with “carotid body tumors” who underwent surgical resection between September 2023 and September 2024 by the same neurosurgical team. The enrollment criteria were as follows: (1) Availability of preoperative multimodal imaging, including carotid artery ultrasound, head and neck computed tomographic angiography (CTA), neck enhanced MRI and neck hr-MRI; (2) Postoperative pathological confirmation of CBTs; (3) The hardness of CBTs was evaluated through the fibrosis ratio in the postoperative Masson staining [6]. Exclusion criteria were as follows: (1) A history of previous ipsilateral CBT surgery or preoperative interventional therapy (e.g., embolization) or radiotherapy; (2) Presence of contraindications to imaging examinations, incomplete imaging data, or poor image quality precluding standardized measurement; (3) Absence of hardness assessment of the CBT. Finally, a total of 82 patients were enrolled in the study.

This study was approved by the institutional review board of Peking University Third Hospital (Approval No. M2023748) and written informed consent was waived due to the anonymized nature of the data.

### 2.2. Clinical Baseline Data

Demographic data (age, gender, arterial blood pressure and BMI), preoperative laboratory examination data (adrenaline and dopamine concentration, C-reactive protein, and the proportion of neutrophils and lymphocytes) and surgical data (operative duration, intraoperative estimated blood loss) were extracted from electronic medical records. Pre- and postoperative nerve injuries were defined as neurological dysfunction (e.g., hoarseness, dysphagia, coughing during swallowing, or Horner’s syndrome) present prior to surgery or developing during the postoperative follow-up period. The follow-up period was uniformly set at 6 months after the surgery. Intraoperative vascular and nerve injury (IVNI) was defined as injury of the internal carotid artery, external carotid artery, and nerves in the surgical area (including the facial nerve, glossopharyngeal nerve, vagus nerve, accessory nerve and their branches, as well as the sympathetic trunk) due to tumor invasion or other causes during surgery.

### 2.3. Imaging Protocol and Data Collection

**Figure 1:**
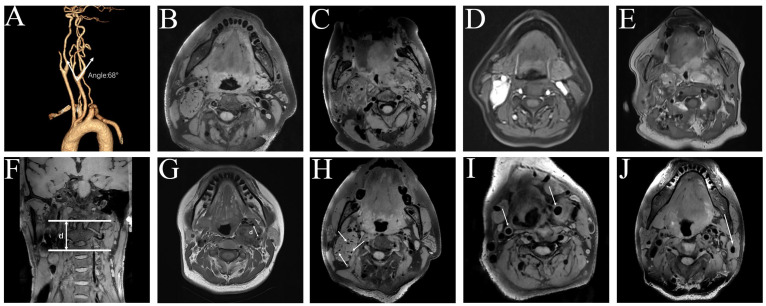
Imaging data collection. The measurement of the bifurcation angles, which was obtained in the reconstructed images of the head and neck CTA (**A**). The signal was relatively homogeneous on T1WI (**B**) and T1WI-CE (**D**) in soft CBTs, but heterogeneous in hard CBTs (**C**,**E**). BD was the distance from the uppermost pole of CBTs (lower straight line) to the apex of the odontoid process of the axis (upper straight line) in the coronal position sequence (**F**). d in subfigure (**F**) means BD. SD was the distance between ICA and ECA (**G**). d in subfigure (**G**) means SD. Newly formed arteries and veins are vessels in CBTs ultimately connected to the carotid artery and internal jugular vein (white arrows) (**H**). PFS was the complete fat structure of the outermost layer of the artery (**I**). The PFS of the right carotid artery was intact, but the PFS of the left carotid artery was eroded by CBTs. The EJV (white arrow) on the left side was dilated (**J**).

Ultrasound echo homogeneity was determined by reviewing preoperative reports, which were verified by at least two senior sonographers. The head and neck CTA was conducted on the same CT scanner (u780, United Imaging Healthcare, Shanghai, China). Iopromide (Bayer Pharma AG, Berlin, Germany) was used as the contrast agent. The sequences acquired on CTA plain scanning using the Siemens 47223 workstation (Siemens Healthcare, Erlangen, Germany) neuro DSA were transferred to AW45-2 (GE Healthcare, Chicago, IL, USA) for analysis. The resulting silhouette sequences were processed using reformatting to obtain bone-removed images, which were saved at rotations of every 15 degrees. In this image processing workstation, the bifurcation angle (BA) was determined by measuring the angle between the internal carotid artery (ICA) and the external carotid artery (ECA) in the reconstructed images of the head and neck CTA (Figure 1A), and the reconstructed image was adjusted on the same axis.

MRI was performed using a 3.0T scanner (uMR780, United Imaging Healthcare, Shanghai, China) with an 8-channel dedicated carotid coil (CAR8, United Imaging Healthcare, Shanghai, China) covering the left and right carotid arteries. The standard hr-MRI protocol included 2D black-blood T1-weighted fast spin-echo and 2D black-blood T2-weighted FSE sequences. Contrast-enhanced T1-weighted (T1WI-CE) images were acquired subsequently following the intravenous injection of a gadolinium-based contrast agent (gadoterate meglumine; Dotarem, Guerbet, Villepinte, France). The raw signal intensity (SI) of CBTs and surrounding tissues, including the ipsilateral sternocleidomastoid muscle (SCM), was obtained via direct measurement on the imaging workstation across different sequences (T1WI, T2WI, and T1WI-CE). To ensure measurement reproducibility, regions of interest (ROIs) were manually drawn on the solid portions of the tumor at its maximum cross-sectional area, deliberately avoiding areas of obvious necrosis, cystic changes, calcification, or major macroscopic blood vessels. For the reference standard, identical ROIs were placed on the uniform belly of the ipsilateral SCM at the same anatomical level. The SCM was selected as the reference tissue due to its immediate anatomical proximity and inclusion within the same coil field-of-view, which minimizes radiofrequency inhomogeneity. To account for inherent scanner variations and coil sensitivity, the raw SI values were mathematically normalized by calculating the CBT-to-SCM ratio. Thus, the CBT/SCM values on T1WI and T2WI were defined as the ratio of the CBT SI to the SCM SI. All measurements were performed independently by two experienced investigators to minimize measurement bias.

The approximate volume of CBTs was calculated by measuring the longest diameters on the coronal, sagittal, and axial sections, multiplying these values, and dividing by 2. BD was defined as the distance from the uppermost pole of CBTs to the base of the skull. This measurement was performed on the coronal hr-MRI sequence as the distance from the level of the tumor’s uppermost pole to the apex of the odontoid process of the axis (Figure 1F). If the CBTs invaded the base of the skull and extended superiorly beyond the level of the odontoid process apex, BD was recorded as a negative value. Separation distance (SD) was defined as the maximal distance between the ICA and ECA lumens, measured in the level where they were farthest apart on hr-MRI (Figure 1G). Newly formed arteries and veins were determined by identifying whether these blood vessels in CBTs ultimately communicated with the carotid artery and internal jugular vein on hr-MRI (Figure 1H). The perivascular fat space (PFS) refers to the layer of fat surrounding the outermost aspect of the ICA and ECA. On T1WI sequences, the PFS appears as a continuous high-signal rim around the blood vessels (Figure 1I, right carotid artery). If the high-signal continuity of T1WI in the outermost layer of ICA or ECA disappeared, it was considered that CBTs had eroded the PFS of ICA or ECA (Figure 1I, left carotid artery). The presence of lymph node hyperplasia and dilation of the external jugular vein (EJV) were assessed by comparing the lymph node characteristics and the EJV diameter on the affected side to those on the healthy side (Figure 1J).

### 2.4. CBTs’ Hardness Definition

**Figure 2:**
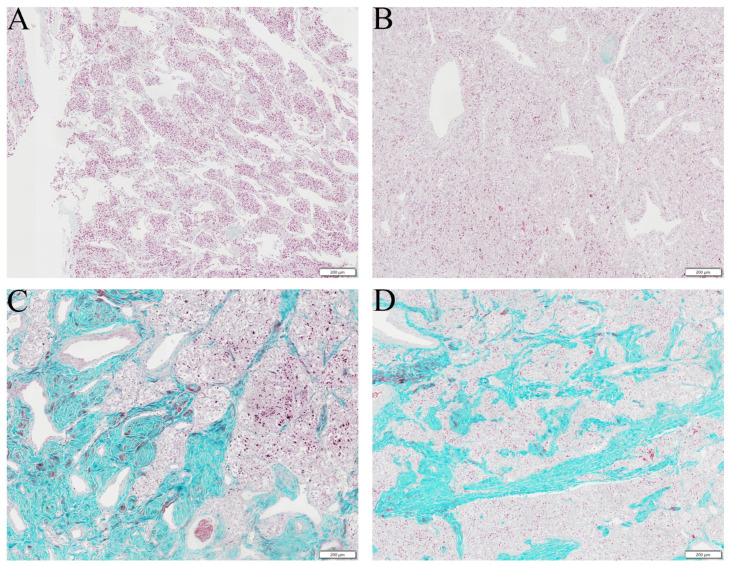
Masson staining of CBTs with different hardness. In soft CBTs, staining results showed a low degree of fibrosis (≤30%, (**A**,**B**)), whereas in hard CBTs, staining results indicated a high degree of fibrosis (>30%, (**C**,**D**)). The blue-stained components are collagen fibers. The purple color in the staining represents the standard background in Masson’s trichrome stain.

CBTs’ hardness was evaluated based on the proportion of fibrosis observed via postoperative Masson staining. Masson staining can specifically display the collagen components in tissues (in blue) and can accurately assess the degree of fibrosis in CBTs. Yao et al. found that CBTs with a higher proportion of fibrous components exhibited greater hardness; therefore, CBTs with a fibrous component proportion of less than 30% were defined as soft, and those with a proportion greater than 30% were defined as hard [6] (Figure 2). To ensure objectivity, the proportion of fibrosis in all Masson-stained sections was evaluated independently by two experienced pathologists who were completely blinded to the patients’ clinical characteristics and preoperative multimodal imaging findings. In cases of discrepancy regarding the classification of tumor hardness (soft vs. hard), a third senior pathologist was consulted to review the histological slides and achieve a final consensus. While we acknowledge that histological fibrosis is not a direct biomechanical measurement, qualitative assessments of tumor hardness (e.g., soft or hard) routinely documented in the surgical records were extracted to further ensure the consistency of the assessment results.

### 2.5. Statistical Analysis and Quality Control

The data of normal distribution were expressed as mean ± standard deviation, and the two-sample independent *t*-test was used for comparison between groups. Non-normally distributed data were expressed as median with interquartile range (IQR), and compared for differences between groups using the Mann–Whitney *U* test. Categorical variables were expressed as frequency and percentage (%). The analysis of differences in categorical variables was conducted using chi-square test or Fisher’s exact test. Variables with *p* < 0.05 in univariate analysis were identified as potential predictors. To ensure the stability of the multivariate logistic regression model and avoid overfitting, the number of included variables was restricted according to the “events per variable” (EPV) rule, focusing on those with the highest clinical relevance and strongest associations. Furthermore, due to the presence of quasi-complete separation observed in certain powerful categorical predictors (specifically, perivascular fat space [PFS] erosion), Firth’s penalized likelihood logistic regression was performed for the multivariate analysis. This approach stabilizes parameter estimates and yields reliable odds ratios (ORs) and 95% confidence intervals (CIs) in the presence of separation. ROC curve analysis was performed to determine the optimal cut-off value of variables and maximize the sensitivity and specificity. Inter-rater reliability between the two investigators was evaluated using the Intraclass Correlation Coefficient (ICC) for continuous variables and Cohen’s kappa for categorical variables, with values > 0.75 indicating excellent agreement. Statistical significance was determined by two-tailed tests with a *p* < 0.05. Statistical analysis was performed using SPSS 26.0 (IBM Corp., Armonk, NY, USA) and Firth’s penalized likelihood logistic regression was executed using R software version 4.4.2 (R Foundation for Statistical Computing, Vienna, Austria) with the “logistf” package.

## 3. Results

### 3.1. Demographic and Clinical Characteristics

A total of 82 patients were enrolled in this study. All 82 CBTs (51 soft and 31 hard) were completely resected, and the hardness evaluated based on the degree of fibrosis were all consistent with the surgical records. The mean age of the 82 patients was 46 ± 13 years, 37 of whom were male. There were no significant differences in age, gender, ABP and BMI between the two groups. Clinically, operative duration was significantly longer for hard CBTs compared to soft CBTs (172 ± 76 min vs. 106 ± 68 min, *p* < 0.001). Although median estimated blood loss was higher in the hard CBTs group (30 [20–100] mL vs. 10 [5–30] mL), this difference did not reach statistical significance (*p* = 0.785). In terms of perioperative complications, the incidence of preoperative (29% vs. 12%, *p* = 0.049) and postoperative (52% vs. 22%, *p* = 0.007) nerve injury, as well as IVNI (26% vs. 4%, *p* = 0.005), were significantly higher in hard CBTs than in soft CBTs (Table 1).

### 3.2. Multimodal Imaging Data Analysis

The inter-rater agreement for all imaging measurements was excellent, with all ICC and Cohen’s kappa values exceeding 0.80. Ultrasound and CTA imaging data showed no significant differences between the two groups. In terms of MRI imaging data, soft CBTs and hard CBTs showed significantly different signal homogeneity on T1WI and T1WI-CE sequences. A significantly higher proportion of soft CBTs exhibited homogeneous signal intensity compared with hard CBTs on T1WI (86% vs. 32%, *p* < 0.001) and T1WI-CE (71% vs. 39%, *p* = 0.004). Soft CBTs exhibited higher SI values on T2WI (795 ± 259 vs. 479 ± 295, *p* < 0.001) and higher CBT/SCM values on T1WI (1.2 ± 0.2 vs. 1.1 ± 0.2, *p* = 0.011) and T2WI (4.6 ± 1.3 vs. 2.3 ± 1.2, *p* < 0.001) compared to hard CBTs. At the same time, the proportions of newly formed draining veins (45% vs. 90%, *p* < 0.001), PFS erosion (16% vs. 90%, *p* < 0.001), lymph node hyperplasia (14% vs. 42%, *p* = 0.004), and EJV dilation (24% vs. 58%, *p* = 0.002) were significantly lower in soft CBTs compared to hard CBTs. Other imaging data, such as ultrasound echo homogeneity, BA on CTA and tumor volume, showed no statistical difference between CBTs with different hardness (Table 2).

**Figure 3:**
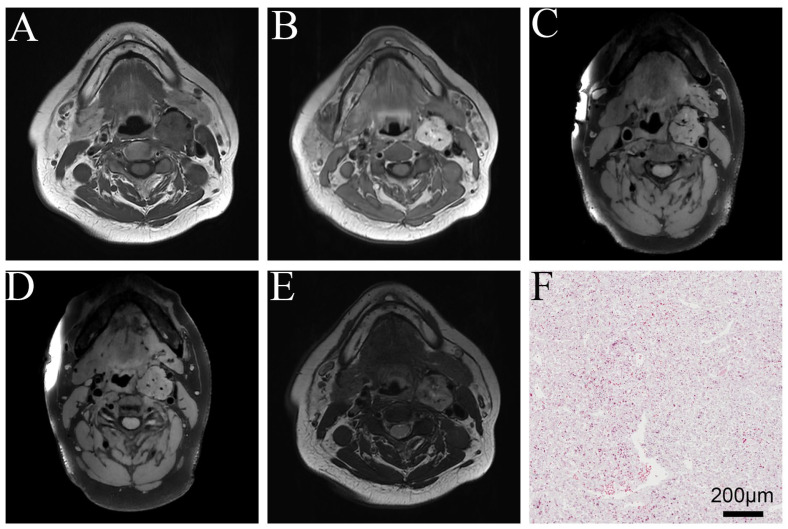
Imaging and pathological results from a case of soft CBTs. The T1WI (**A**) and T1WI-CE (**B**) signal intensity was relatively homogeneous in this case. The PFS was still intact (**C**) and few new vessels formed (**D**). The T2WI SI value (**E**) was relatively high. Pathological staining (**F**) indicated a relatively low collagen content.

**Figure 4:**
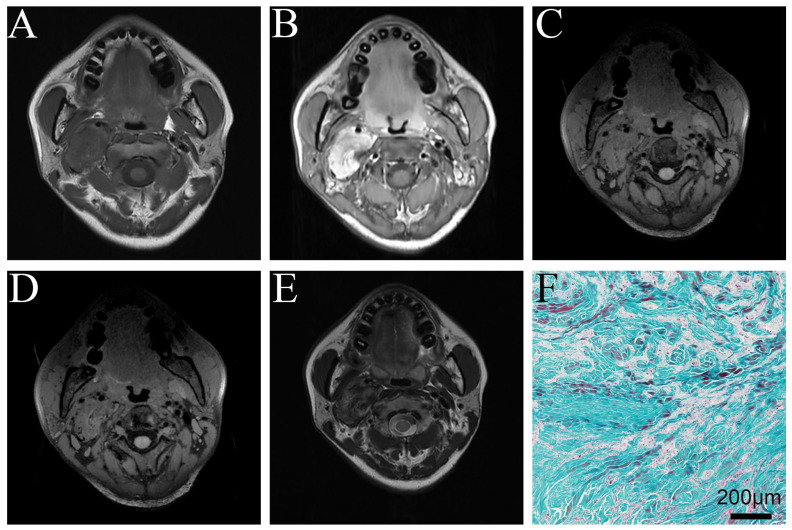
Imaging and pathological results from a case of hard CBTs. The T1WI (**A**) and T1WI-CE (**B**) signal intensity was relatively heterogeneous in this case. The PFS was eroded (**C**) and more new vessels formed (**D**). The T2WI SI value (**E**) was relatively low. Pathological staining (**F**) indicated a relatively high collagen content.

Univariate logistic regression was performed for imaging variables with statistical differences, and all of these variables were associated with the hardness of CBTs. To comprehensively evaluate the predictive value of imaging, three variables representing different pathological dimensions were selected for the multivariate model: CBT/SCM on T2WI (representing internal tissue signal and composition), PFS erosion (representing local invasiveness), and lymph node hyperplasia (reflecting regional immune response and potential aggressiveness). CBT/SCM value on T2WI (OR 0.329; 95% CI 0.151–0.591; *p* < 0.001) and PFS erosion (OR 19.2; 95% CI 4.390–115.884; *p* < 0.001) remained independent predictors of CBTs’ hardness. However, lymph node hyperplasia did not reach statistical significance as an independent predictor in this model (Table 3) (Figure 3 and Figure 4).

### 3.3. ROC Curve of CBT/SCM Value on T2WI

**Figure 5:**
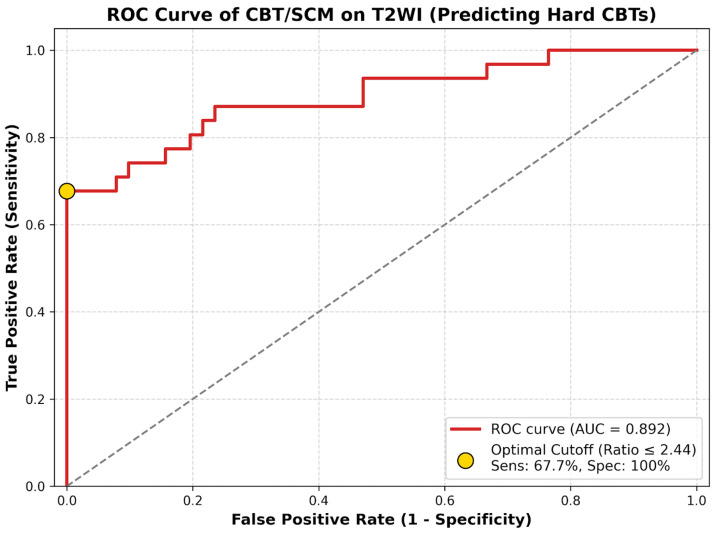
The ROC curve of CBT/SCM value on T2WI. The dashed grey diagonal line represents the reference line for a random classifier (AUC = 0.5).

The analysis of ROC curves (Figure 5) showed that the optimal cutoff value of the CBT/SCM ratio on T2WI for predicting hard CBTs was 2.44 (AUC = 0.892; 95% CI: 0.814–0.969; *p* < 0.001). A CBT/SCM ratio ≤ 2.44 predicted a hard tumor with a sensitivity of 67.7%, a specificity of 100%, a positive predictive value (PPV) of 100%, and a negative predictive value (NPV) of 83.6%.

## 4. Discussion

### 4.1. Hardness and Surgical Complexity

Our study found that compared to soft CBTs, hard CBTs were associated with longer operative duration and higher rates of perioperative complications, indicating greater treatment difficulty. Our present study highlights the critical role of preoperative multimodal imaging in evaluating the hardness of CBTs and its implications for surgical planning. Our findings demonstrate that specific imaging biomarkers, particularly the CBT/SCM value on T2WI and PFS erosion, are independently associated with tumor hardness, which could serve as a crucial component of overall surgical complexity. Regarding the definition of tumor hardness, we adopted the 30% fibrosis proportion cutoff established by Yao et al. [6]. In our specific cohort, this histological threshold robustly correlated with prolonged operative durations and increased neurovascular injury rates, further validating its practical clinical relevance. Importantly, surgical perception of hardness might be the composite of both intrinsic tumor hardness (fibrosis) and local invasiveness (e.g., perivascular adherence and inflammatory changes). Our proposed imaging markers efficiently capture both dimensions: the CBT/SCM ratio on T2WI reflects intrinsic composition, while PFS erosion indicates local perivascular invasiveness. However, we acknowledge that categorizing tumor hardness into a binary system (soft vs. hard) is a simplification of a continuous biological spectrum. Future prospective studies correlating this histological threshold with direct intraoperative biomechanical mapping (e.g., elastography or physical durometer measurements) are warranted to universally validate this cutoff.

### 4.2. Predictive Value of CBT/SCM Ratio on T2WI

The application of MRI for tissue hardness assessment is well-established in other medical fields, though methodologies differ. Magnetic resonance elastography (MRE) has been widely used in the diagnosis of liver fibrosis since it was first proposed in 1995 [8], and is now also playing a role in the field of neurosurgery [9,10]. MRE directly quantifies tissue stiffness by measuring shear wave propagation. While highly accurate, MRE requires specialized sequences and hardware, limiting its accessibility for routine CBT evaluation. Apparent diffusion coefficient (ADC) values reflect cellular density, indirectly inferring stiffness. ADC values have also been applied to assess the hardness of pancreatic tissue [11]. However, ADC lacks specificity in highly vascularized tumors like CBTs due to the confounding effects of microvascular flow [12]. While quantitative MRI signal intensity ratios have been well-validated in the characterization of other neuroendocrine neoplasms [13,14] (such as adrenal pheochromocytomas) and evaluating histological subtypes in intracranial malignant tumors [15], their specific application in evaluating the hardness of CBTs has rarely been reported in prior literature. Our study addresses this gap by translating this established quantitative radiological concept to the preoperative assessment of CBTs. Compared to these advanced sequences, the CBT/SCM value on T2WI offers a simpler, clinically feasible alternative. It circumvents the need for advanced sequences like MRE or ADC, making it adaptable to standard MRI protocols. This is particularly advantageous in resource-limited settings or for CBTs located in anatomically complex regions like the carotid bifurcation, where motion artifacts may degrade elastography accuracy [16]. The CBT/SCM value on T2WI emerged as a robust imaging biomarker for distinguishing soft and hard CBTs. A lower CBT/SCM value on T2WI in hard CBTs likely reflects reduced extracellular fluid content and increased fibrous tissue density, which restricts the mobility of water, thereby shortening the T2 relaxation time, consistent with the histopathological characteristics of hard tumors [17,18]. This biomarker not only aids in predicting tumor hardness but also correlates with prolonged operative duration, as hard CBTs often require meticulous dissection to avoid neurovascular injury. Clinically, preoperative identification of low CBT/SCM value on T2WI could prompt surgeons to allocate additional resources, such as intraoperative neuromonitoring or vascular reconstruction teams, to mitigate complications. ROC curve analysis identified an optimal cutoff value of ≤2.44 for the CBT/SCM ratio on T2WI, demonstrating a specificity of 100% and a positive predictive value (PPV) of 100% for predicting hard CBTs. Clinically, this high specificity offers substantial utility as a confirmatory indicator; a ratio below this threshold reliably predicts a fibrotic, surgically complex lesion with a negligible risk of false positives. Thus, this biomarker provides an objective rationale for proactively mobilizing advanced surgical resources—such as intraoperative neuromonitoring and vascular reconstruction teams—optimizing patient safety while mitigating the healthcare costs associated with unwarranted surgical preparation. Conversely, the moderate sensitivity (67.7%) and negative predictive value (83.6%) suggest that a ratio > 2.44 cannot definitively exclude tumor hardness. Crucially, this implies that approximately 33% of histologically hard tumors would be misclassified as “soft” (false negatives). The clinical consequence of missing these hard tumors is severe: it risks leaving surgical teams under-prepared for unexpectedly difficult dissections and catastrophic neurovascular injuries. Therefore, this ratio must never be used in isolation to rule out complex dissections. Therefore, baseline vigilance for neurovascular complexities remains essential, and preoperative accuracy may be further enhanced by integrating this ratio with other morphological markers of local invasiveness, such as PFS erosion.

### 4.3. The Role of PFS Erosion and Invasiveness

PFS erosion is another independent predictor of CBTs’ hardness. Tumors exhibiting PFS erosion may represent aggressive lesions that infiltrate surrounding adipose tissue, potentially complicating surgical margins and increasing the risk of residual disease [19]. The multivariate analysis (OR 19.2; *p* < 0.001) highlights its strong association with hard CBTs. PFS erosion serves as a reliable indicator of tumor hardness, reflecting increased local aggressiveness and a higher risk of surgical complications. In various malignancies (e.g., pancreatic adenocarcinoma, renal cell carcinoma), adipose tissue infiltration is linked to advanced tumor stages, metastasis, and poor prognosis [20,21]. Similarly, in CBTs, PFS erosion indicates encroachment into the carotid artery, even the vagus nerve or sympathetic chain, increasing intraoperative dissection challenges and potential postoperative recurrence and complication risks. While our study primarily focuses on macroscopic imaging findings, we speculate that PFS erosion might also be related to alterations in the local microenvironment, influencing tumor progression. Adipocytes release free fatty acids and adipokines, which may activate tumor-associated macrophages and stimulate cytokines secretion, fostering angiogenesis [22,23]. This theoretical mechanism could explain the higher prevalence of newly formed draining veins in hard CBTs observed in our study. Its clinical utility extends beyond preoperative assessment, offering novel insights into CBT biology and guiding precision therapeutic strategies.

While CBT/SCM value on T2WI and PFS erosion are the most significant predictors in our study, other imaging features are also worth discussion. The presence of newly formed draining veins and lymph node hyperplasia in hard CBTs suggests heightened angiogenic activity and immune response [24], which may reflect tumor aggressiveness and local tissue invasion, ultimately contributing to neurovascular damage. Although lymph node hyperplasia did not retain independent significance in the multivariate model, its preoperative identification remains clinically valuable. It alerts surgeons to potential surgical complexity and informs perioperative planning, such as the need for concurrent lymph node dissection. Similarly, T1WI and T1WI-CE homogeneity, though not independent predictors, offered preliminary insights into tumor microstructure, warranting further investigation with advanced sequences like diffusion-weighted imaging.

### 4.4. Clinical Significance and Risk Control

Due to the invasion and adhesion of hard CBTs to surrounding carotid vessels and nerves, their resection is associated with greater surgical difficulty and a higher risk of perioperative complications [25]. Preoperative assessment of tumor hardness holds significant clinical value in formulating appropriate treatment strategies and preparing for potential vascular and nerve repair. In clinical practice, the identification of a low CBT/SCM ratio on T2WI or PFS erosion should prompt enhanced perioperative preparation. This includes more detailed patient counseling, informing patients about a higher risk of perioperative complications, anticipating difficult anatomical dissection due to adhesions, utilizing intraoperative nerve monitoring techniques, and preparing for the possibility of vascular reconstruction.

### 4.5. Limitations

Our study has several limitations. First, the retrospective design and modest sample size from a single center inherently limit the generalizability of our results. Given the extreme rarity of CBTs, a formal a priori power analysis was not feasible, though we adhered to the EPV rule to mitigate overfitting. Furthermore, excluding patients with preoperative embolization restricts our model’s real-world applicability strictly to treatment-naive tumors. Additionally, using the SCM as an internal reference is inherently imperfect, as its baseline signal may fluctuate with individual muscle mass or fatty infiltration. Second, the optimal cutoff value for the CBT/SCM ratio on T2WI (2.44) was derived and evaluated within the exact same cohort. Deriving and evaluating the cutoff value within the same cohort risks overestimating the diagnostic performance, underscoring the absolute necessity for future independent, external validation cohorts. Third, although we utilized Firth’s penalized likelihood logistic regression to address the quasi-complete separation issue and mathematically stabilize our multivariate model, the confidence interval for PFS erosion remains relatively wide due to the limited absolute number of hard CBTs. Furthermore, our assessment of CBT hardness relied on the proportion of fibrosis rather than direct biomechanical measurements (e.g., intraoperative elastography or elastic modulus). Although this histological threshold matched our intraoperative qualitative records, assuming fibrosis equates to physical hardness remains a limitation. Future prospective, multi-center studies incorporating direct physical hardness data are required to rigorously validate these findings.

## 5. Conclusions

Our findings suggest that the CBT/SCM value on T2WI and PFS erosion show promising potential as independent imaging biomarkers for evaluating CBT hardness. These parameters may facilitate the development of preoperative risk prediction, which could aid in reducing perioperative risks and improving outcomes. However, considering the retrospective, single-center design and the absence of direct biomechanical hardness assessment, these results require further prospective validation. This study provides a valuable theoretical foundation for advancing precision surgery for CBTs and underscores the critical role of multimodal imaging in preoperative evaluation.

## Figures and Tables

**Table 1:** Demographic and clinical characteristics of all patients.

Variable	All Patients (*n* = 82)	Soft CBTs (*n* = 51)	Hard CBTs (*n* = 31)	*p*-Value
Age, year	46 ± 13	46 ± 13	45 ± 14	0.630
Gender, Male	37 (45%)	25 (49%)	12 (39%)	0.363
BMI, kg/m^2^	24 ± 3	24 ± 4	23 ± 3	0.225
ABP, mmHg	90 ± 4	90 ± 4	90 ± 4	0.799
CRP, mg/L	2.1 [0.8–6.0]	1.6 [0.7–5.7]	3.2 [0.8–6.4]	0.627
Neutrophils, %	64 ± 10	62 ± 10	66 ± 9	0.115
Lymphocytes, %	26 ± 6	27 ± 7	26 ± 5	0.503
Adrenaline, pg/mL	5.3 ± 2.4	5.1 ± 2.2	5.5 ± 2.7	0.466
Dopamine, pg/mL	22 ± 4	23 ± 4	21 ± 4	0.108
Operative Duration, min	131 ± 77	106 ± 68	172 ± 76	<0.001
Blood loss, mL	20 [9–50]	10 [5–30]	30 [20–100]	0.785
Pre-op NI	15 (18%)	6 (12%)	9 (29%)	0.049
IVNI	10 (12%)	2 (4.0%)	8 (26%)	0.005
Post-op NI	27 (33%)	11 (22%)	16 (52%)	0.007

Note: Normally distributed data were expressed as mean ± standard deviation, non-normally distributed data were expressed as median [interquartile range (IQR)]; categorical variables were expressed as *n* (%). Abbreviation: ABP: arterial blood pressure; CRP: C-reactive protein; Pre-op NI: preoperative nerve injury; IVNI: intraoperative vascular and nerve injury; Post-op NI: postoperative nerve injury.

**Table 2:** Univariate analysis of multimodal imaging for CBTs.

Variable	Soft CBTs (*n* = 51)	Hard CBTs (*n* = 31)	*p*-Value
Ultrasound echo, homogeneous	14 (27%)	7 (23%)	0.624
BA, degree	82 ± 14	84 ± 16	0.470
T1WI, homogeneous	44 (86%)	10 (32%)	<0.001
T1WI, SI	577 ± 185	564 ± 201	0.752
T1WI-CE, homogeneous	36 (71%)	12 (39%)	0.004
T1WI-CE, SI	1565 ± 459	1380 ± 453	0.079
CBT/SCM on T1WI	1.2 ± 0.2	1.1 ± 0.2	0.011
T2WI, SI	795 ± 259	479 ± 295	<0.001
CBT/SCM on T2WI	4.6 ± 1.3	2.3 ± 1.2	<0.001
Tumor volume, cm^3^	8 [4–16]	17 [10–36]	0.083
BD, mm	22 ± 14	15 ± 21	0.093
SD, mm	16 ± 5	17 ± 7	0.284
Artery, newly formed	46 (90%)	30 (97%)	0.267
Draining vein, newly formed	23 (45%)	28 (90%)	<0.001
PFS, erosion	8 (16%)	28 (90%)	<0.001
Lymph node, hyperplasia	7 (14%)	13 (42%)	0.004
EJV, dilation	12 (24%)	18 (58%)	0.002

Note: Normally distributed data were expressed as mean ± standard deviation, non-normally distributed data were expressed as median [interquartile range (IQR)]; categorical variables were expressed as *n* (%). Abbreviation: BA: bifurcation angles of ICA and ECA; SI: Signal Intensity; SCM: sternocleidomastoid muscle; BD: the distance from the uppermost pole of CBTs to the base of the skull; SD: separation distance between ICA and ECA; PFS: perivascular fat space; EJV: external jugular vein.

**Table 3:** Firth’s Penalized Likelihood Logistic Regression analysis of multimodal imaging for CBTs.

Variable	Univariate Analysis		Multivariate Analysis	
	OR (95% CI)	*p*-Value	OR (95% CI)	*p*-Value
T1WI, homogeneous	0.082 (0.027–0.229)	<0.001	-	-
T1WI-CE, homogeneous	0.272 (0.105–0.676)	0.005	-	-
CBT/SCM on T1WI	0.029 (0.001–0.452)	0.012	-	-
T2WI, SI	0.996 (0.993–0.998)	<0.001	-	-
CBT/SCM on T2WI	0.270 (0.146–0.438)	<0.001	0.329 (0.151–0.591)	<0.001
Draining vein, newly formed	9.875 (3.179–40.229)	<0.001	-	-
PFS, erosion	41.672 (12.274–185.136)	<0.001	19.2 (4.390–115.884)	<0.001
Lymph node, hyperplasia	4.330 (1.560–12.858)	0.005	3.076 (0.476–23.133)	0.239
EJV, dilation	4.330 (1.710–11.479)	0.002	-	-

Note: Tables without data indicate variables not included in the multivariate model due to collinearity or to ensure model parsimony and stability based on the events-per-variable (EPV) rule. Multivariate analysis was performed using Firth’s penalized likelihood logistic regression to account for quasi-complete separation.

## Data Availability

The data and materials are available from the corresponding authors on reasonable request.

## Abbreviations

The following abbreviations are used in this manuscript:

ADC	Apparent Diffusion Coefficient
BA	Bifurcation Angles
CBTs	Carotid Body Tumors
CT	Computed Tomographic
CTA	Computed Tomographic Angiography
ECA	External Carotid Artery
EJV	External Jugular Vein
EPV	Events Per Variable
ICA	Internal Carotid Artery
IVNI	Intraoperative Vascular and Nerve Injury
IQR	Interquartile Range
MRI	Magnetic Resonance Imaging
MRE	Magnetic Resonance Elastography
NPV	Negative Predictive Value
SCM	Sternocleidomastoid Muscle
SD	Separation Distance
SI	Signal Intensity
PFS	Perivascular Fat Space
PPV	Positive Predictive Value
